# Revolutionizing Dentistry: Preclinical Insights and Future Applications of mRNA Vaccines in Dentistry—A Narrative Review

**DOI:** 10.3390/dj13020079

**Published:** 2025-02-13

**Authors:** Luciana Koren, Andro Koren, Robert Likić, Tomislav Katanec

**Affiliations:** 1 School of Medicine, University of Zagreb, 10000 Zagreb, Croatia; koren.luciana@gmail.com (L.K.); androkoren54@gmail.com (A.K.); 2 Unit for Clinical Pharmacology, Department of Internal Medicine, Clinical Hospital Centre Zagreb, 10000 Zagreb, Croatia; rlikic@kbc-zagreb.hr; 3 Department of Oral Surgery, School of Dental Medicine Zagreb, Clinical Hospital Centre Zagreb, 10000 Zagreb, Croatia

**Keywords:** dentistry, mRNA vaccines, periodontal disease, oral health, dental caries, immune modulation, implantology, regenerative medicine

## Abstract

**Background:** Recent advances in mRNA vaccine technology, accelerated by the global COVID-19 pandemic, have generated significant interest in their applications beyond infectious diseases. Dentistry has emerged as a promising field for exploring the potential of mRNA-based therapies in preventing and treating oral diseases. **Objectives:** This narrative review aims to evaluate the current status of mRNA vaccine development and its preclinical applications in oral health, focusing on periodontal disease, dental caries, regenerative medicine, implantology, and oral cancer. **Methods:** The review synthesizes findings from preclinical studies, including research conducted in animal models and in vitro, to assess the potential of mRNA-based therapies to modulate immune responses and promote tissue regeneration in the oral cavity. Clinical trials were only mentioned in the context of broader areas of mRNA vaccine implementation such as oncology and immunotherapy. **Results:** The preclinical studies highlight the capacity of mRNA vaccines to enhance the body’s immune response and facilitate tissue repair processes. Despite these promising results, challenges persist in delivering mRNA vaccines effectively within the complex oral environment. These challenges include vaccine stability, delivery mechanisms, and the modulation of immune responses. **Conclusions:** While mRNA vaccines offer significant promise for revolutionizing oral health care, they face notable limitations concerning safety, efficacy, and clinical feasibility. Overcoming these obstacles through further research is essential to unlock their full translational potential and ensure their safe and effective integration into dental practice.

## 1. Introduction

**Figure 1:**
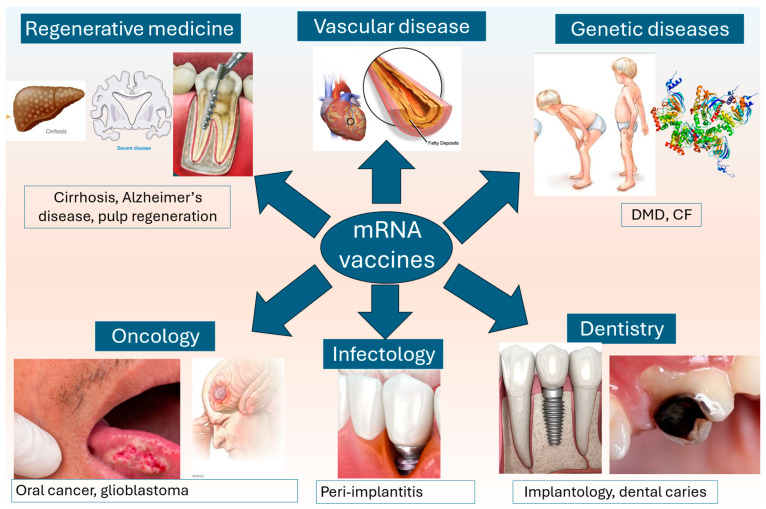
The potential of mRNA vaccines in different fields of medicine owing to their anti-inflammatory, immunomodulatory, antitumor, and regenerative effects.

mRNA vaccines have proven to exhibit great potency amid the recent COVID-19 pandemic [1]. Nevertheless, one should not stop at just recognizing the significant effects demonstrated in the field of infectious diseases and epidemiology [2]. Such vaccines have been proven to play a role in the complex immunopathogenesis of numerous conditions, displaying anti-inflammatory, immunomodulatory, antitumor, and regenerative effects [3,4,5] (Figure 1). Despite these advancements, their application in dentistry is still in its early stages [6]. Research has primarily focused on the potential for mRNA vaccines to address common oral health issues like periodontal disease [7], implantology [8], dental caries [9], and oral cancer [10]. However, there are significant gaps in understanding how mRNA vaccines can be effectively tailored to the unique environment of the oral cavity [11]. Key challenges include navigating microbial interactions, mitigating immune response risks, ensuring long-term safety, and addressing cost barriers to promote equitable access. Additionally, optimizing delivery methods and enhancing the ability to modulate local immune responses for effective tissue regeneration are critical [12]. This narrative review critically examines preclinical studies on mRNA vaccines in dentistry, assessing their potential to revolutionize oral health care [13]. Through a comprehensive analysis of the existing research, we aim to clarify the current knowledge, highlight gaps, and propose future research directions [14]. Ultimately, we seek to advance mRNA vaccines as a promising therapeutic option in dentistry, addressing both the promise and the challenges of this emerging field [15].

## 2. Basic Principles

### 2.1. mRNA Vaccines in Dentistry: Overview and Potential

The science behind mRNA vaccines and their production and application is advancing at an unprecedented pace, largely due to promising results in numerous preclinical studies across a broad spectrum of medical fields [16,17,18,19]. Notably, mRNA-based therapies are being recognized as potential solutions for a range of conditions that pose a significant economic burden, such as cancer, heart failure, immunological diseases [20], and even rare genetic diseases [21]. Their unique appeal lies not only in their versatility but also in the relatively low-cost and straightforward production methods, coupled with an application process that is less complex and associated with relatively few adverse effects [22]. mRNA is directly delivered to the cytoplasm using nanoparticle carriers, protecting it from degradation and enabling efficient cell uptake. Since translation occurs in the cytoplasm, there is no risk of foreign DNA integration into the genome, and without the need for viral vectors, the risk of insertional mutagenesis and autoimmune reactions is significantly reduced [23]. Although these advances are encouraging, the application of mRNA vaccines in dentistry is still in its early stages, but it holds significant potential for the future [24]. mRNA technology offers an exciting opportunity to address dental pathologies such as periodontal disease, dental caries, and oral cancers—conditions that have been proven challenging to treat effectively [25]. One of the key advantages of mRNA vaccines is their ability to modulate immune responses and stimulate tissue regeneration [26]. For instance, in oral lichen planus (OLP), mRNA encoding immunomodulatory proteins such as interleukin-10 (IL-10) and transforming growth factor-beta (TGF-β) could help regulate the T-cell-mediated immune response, reducing inflammation in OLP, while mRNA encoding growth factors like epidermal growth factor (EGF) and fibroblast growth factor (FGF) could stimulate cell proliferation and tissue repair in the damaged oral mucosa [27].

### 2.2. Challenges of mRNA Vaccine Delivery in Dentistry

Despite the promising prospects of mRNA vaccines, several challenges must be addressed before they can be applied in clinical dentistry. One of the primary concerns is the stability of mRNA within the dynamic environment of the oral cavity [28]. The constant presence of saliva, varying pH levels, and the microbial load of the mouth present significant obstacles to mRNA stability [29]. Optimizing delivery mechanisms to ensure that mRNA effectively targets oral tissues is crucial for success [30]. Furthermore, balancing immune activation is vital to avoid excessive inflammation, which could lead to the exacerbation of existing conditions [31]. While preclinical studies have shown that mRNA vaccines can modulate immune responses and support tissue regeneration, clinical application in dentistry requires further research [32]. Identifying dental-specific antigens and refining delivery systems for the oral cavity are key areas for investigation to improve the effectiveness of mRNA vaccines in this field [33].

### 2.3. Risks, Ethical Considerations, and Future Directions

As with any emerging technology, the potential risks associated with mRNA vaccines in dentistry must be thoroughly evaluated. While mRNA vaccines have shown promise in other clinical trials, there is still concern about local inflammation and unintended immune responses when applied in the oral cavity [34]. Comprehensive preclinical (in vitro and animal models) and clinical studies focused on dental conditions are essential to determine the safety and efficacy of these vaccines [35]. Moreover, ethical considerations, such as patient safety, access to new treatments, and the use of regenerative technologies for non-medical cosmetic procedures, need careful attention [36]. As research in this area progresses, it will be crucial to ensure that mRNA vaccines are both safe and effective for widespread dental use, paving the way for their integration into routine dental care [37].

## 3. Head and Neck Cancer

### 3.1. mRNA Vaccines in HPV-Related Head and Neck Cancer

**Figure 2:**
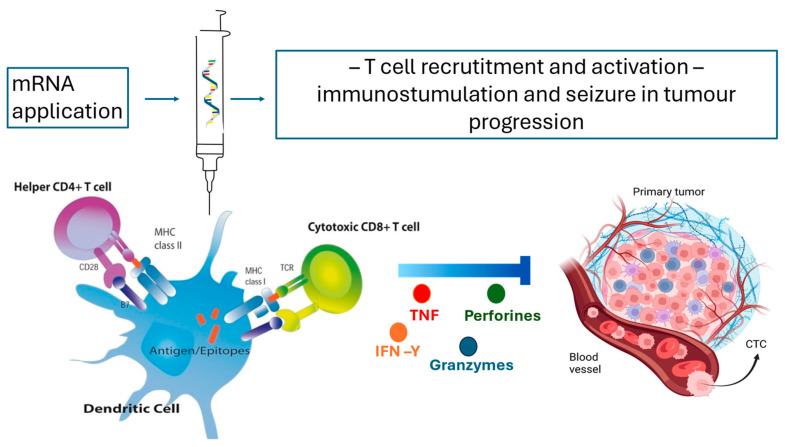
The future of immunotherapy and personalized medicine in oncology using mRNA vaccines—the application of mRNA molecules causes the translation and synthesis of a desired protein which is then expressed on an APC using MHC I and MHC II molecules. Co-stimulation with CD28 and TCR induces a strong CD4 and CD8 T-cell response, resulting in an immunostimulatory antitumor effect and the slowing down of tumor progression.

RNA vaccines have recently gained attention in clinical research for their potential to treat head and neck cancers, particularly those associated with human papillomavirus (HPV) infections [38]. In preclinical studies, several types of mRNA vaccines are being explored, including self-amplifying mRNA, unmodified non-replicating mRNA, and nucleoside-modified non-replicating mRNA vaccines [39]. These vaccines induce the production of oncogenic proteins, such as Gde7, which triggers an immune response that targets tumor-specific antigens [40] (Figure 2). Notably, the activation of CD8+ T lymphocytes has shown promise in halting tumor progression, surpassing the effectiveness of earlier DNA-based Gde7 vaccines [41]. However, it is important to recognize that cancer immunotherapies often show early promise but can face challenges with reproducibility and efficacy across different models [42].

The delivery of mRNA vaccines in these studies is primarily conducted using two main methods: direct injection and ex vivo dendritic cell (DC) loading [43]. Direct injection offers a faster, more cost-effective approach, but it faces the challenge of RNA degradation by endogenous RNases in the body [44]. This can be mitigated by encapsulating the mRNA in lipid nanoparticles (LNPs) or cationic complexes [45]. These delivery systems improve stability, enhance cellular uptake, and support the immune activation necessary for vaccine efficacy [46]. On the other hand, ex vivo dendritic cell (DC) loading with mRNA vaccines offers the advantage of precise control over antigen presentation and a potentially enhanced immune response, but it is limited by the complexity, time consumption, and high costs associated with isolating, modifying, and reintroducing DCs into the patient.

### 3.2. Expanding mRNA Vaccines for Non-HPV-Related Head and Neck Cancers

Although lipid nanoparticles (LNPs) have proven effective in enhancing the stability and uptake of mRNA vaccines, their use comes with some concerns. These include potential toxicity, unintended immune responses, and difficulties in achieving targeted delivery [47]. These risks are common to nanoparticle-based delivery systems, and careful exploration is needed to optimize these methods for use in head and neck cancer treatments [48]. The success of mRNA vaccines depends on balancing efficacy with safety and addressing these concerns through further research [49].

While the current research primarily focuses on HPV-related head and neck cancers, the potential to extend mRNA vaccines to treat non-HPV-related tumors represents a significant opportunity [50].

Although immune responses in murine models have shown promise, further studies are necessary to evaluate the magnitude, durability, and quality of these responses [51]. Long-term studies are crucial for determining the clinical relevance of these preclinical findings [52]. In conclusion, mRNA vaccines show considerable potential in the treatment of head and neck cancers, especially those related to HPV [53]. Addressing the challenges related to delivery, immune response evaluation, and the inclusion of non-HPV-related tumors will be key to advancing mRNA vaccines from preclinical models to clinical applications.

## 4. Regenerative Medicine

### 4.1. mRNA Vaccines in Regenerative Medicine: Mechanisms and Approaches

mRNA vaccines are also attracting attention and starting to scrape the surfaces of the promising future of regenerative medicine by encoding various growth factors that, in turn, cause cellular reprogramming and differentiation [54]. A major advantage of mRNA therapies is their safety profile, which avoids the genomic integration risks associated with traditional gene therapies, garnering increased attention [55]. In recent years, two methods have been developed: the mRNA-induced differentiation of induced pluripotent stem cells (iPSCs) and the direct reprogramming of somatic cells [56].

**Figure 3:**
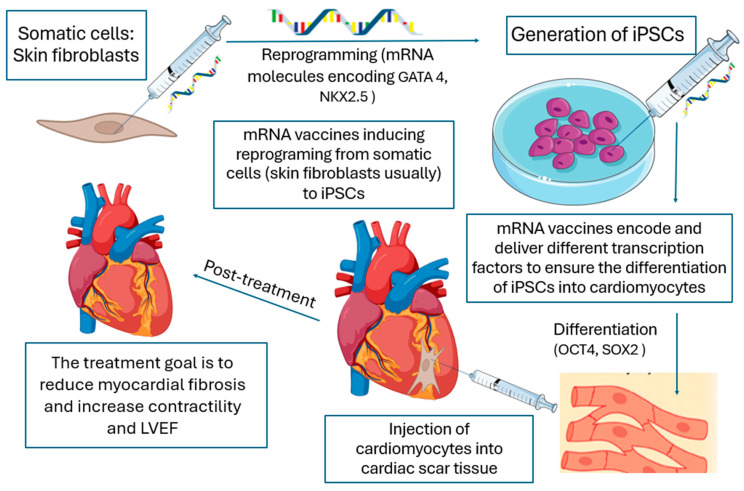
The principles of applying mRNA vaccines in regenerative medicine, as relevant to dentistry, are consistent across medical fields. The cardiac model of myocardial fibrosis illustrates how mRNA vaccines promote iPSC generation, reprogramming, and differentiation to regenerate functional cardiomyocytes, improving heart contractility and left ventricular ejection fraction (LVEF). The goal is to restore functional parenchyma. mRNA vaccines deliver key transcription factors, including OCT4 and SOX2 to facilitate differentiation, and GATA4 and NKX2.5 to promote reprogramming.

The iPSC-based method involves reprogramming somatic cells into a pluripotent state, followed by differentiation into target cell types. While effective, this approach risks tumorigenesis due to residual undifferentiated cells [57]. Conversely, direct reprogramming bypasses the pluripotent stage, converting somatic cells directly into functional cells. This method is safer but faces challenges in efficiency and scalability for clinical applications [58] (Figure 3). mRNA therapies have demonstrated broad regenerative capacity across medical fields, with VEGF-A mRNA aiding cardiac repair and dystrophin-targeted therapies advancing muscular regeneration in conditions like Duchenne muscular dystrophy [59,60].

### 4.2. mRNA-Based Regenerative Therapies in Dentistry: Opportunities and Challenges

In dental care, mRNA regenerative therapies have exhibited potential in implantology and bone grafting. Itaka et al. showed that mRNA encoding Runx2, a transcription factor for osteogenesis, and VEGF, an angiogenesis-promoting factor, synergistically enhanced bone regeneration in rats with jawbone defects [61]. Runx2 induces osteoblast differentiation, while VEGF promotes angiogenesis, together creating a microenvironment conducive to bone repair while markers like osteopontin and osteocalcin were significantly upregulated, supporting osteogenic differentiation [62,63]. Similar studies by Zhang et al. (2023) and Xu Q et al. (2019) corroborated these findings, highlighting the combined action of Runx2 and VEGF in creating optimal environments for bone healing [64,65]. While the results by Zhang et al. (2023) were promising, the study was limited to a preclinical trial involving a sample of 30 rats [66]. Future steps should include trials on larger animal models to validate the findings and optimization for subsequent clinical trials in humans. Furthermore, advances in delivery technologies, such as lipid nanoparticles (LNPs), require further optimization to balance stability, efficiency, and minimal immune activation [67]. Regulatory and ethical considerations play a pivotal role in transitioning mRNA regenerative medicine from research to clinical practice. Key priorities include ensuring long-term safety, mitigating immune response risks, and addressing cost barriers to promote equitable access [68]. Advancing regenerative medicine will require integrating multidisciplinary efforts to overcome these challenges and fully harness the potential of mRNA-based therapies.

## 5. Implantology

### 5.1. The Promise of mRNA Vaccines in Dental Implantology

The application of mRNA vaccines in dental implantology offers a significant opportunity to enhance therapeutic outcomes and improve patient recovery by targeting specific molecular pathways, enabling immune responses, and promoting healing that was first observed in orthopedics joint implants [69]. However, translating these promising results from orthopedic applications to dental implantology requires addressing the oral cavity’s unique conditions, such as microbial flora and bone quality [70]. mRNA vaccines aid in reducing postoperative inflammation by modulating immune responses and promoting the production of anti-inflammatory cytokines, which prevent excessive immune activation at the implant site [71]. The localized expression of VEGF enhances angiogenesis, ensuring adequate blood supply, while BMPs (Bone Morphogenetic Proteins), particularly BMP-2, stimulate osteoblast differentiation and promote mineralization, accelerating the healing process [72]. This combined action fosters osteointegration, where new bone tissue forms around the implant, improving long-term stability, reducing the risk of implant failure, and minimizing infection rates [73]. Such potent anti-inflammatory, immunomodulatory, and regenerative effects open new doors for RNA vaccines in innovating dental implantology [74].

### 5.2. Research Progress and Challenges in mRNA Dental Applications

Recent preclinical studies offer promising evidence for the application of mRNA vaccines in dental implantology. For instance, research by Liu et al. (2023) found that mRNA encoding BMP-2 enhanced osteogenesis in periodontal ligament stem cells, suggesting the potential for mRNA vaccines to improve dental implant integration [75]. Similarly, studies by Zhou et al. (2021) showed that mRNA vaccines encoding BMP-2 accelerated healing and improved implant stability in a rat model of dental implant failure [76]. These findings support the use of mRNA vaccines to improve implant success, particularly in cases with compromised bone quality or delayed healing.

However, several challenges remain. A major concern is the risk of immune overactivation, which could lead to excessive inflammation and interfere with healing [77]. Additionally, ensuring the stability and targeted delivery of mRNA vaccines in the oral cavity is critical, as the oral environment presents challenges like fluctuating temperatures and moisture. Lipid nanoparticles (LNPs), which have been used in other applications, show promise, but further research is needed for optimization. To optimize lipid nanoparticles (LNPs) for dental applications, the particle size should be carefully controlled between 50 and 150 nm to facilitate efficient cellular uptake while avoiding clearance by the reticuloendothelial system. Additionally, modifying the lipid composition, such as using ionizable lipids and PEGylated lipids, can enhance the stability of the LNPs in the oral cavity and improve their ability to release mRNA at the target site, ensuring effective delivery to oral tissues or bone for dental implant regeneration [78].

### 5.3. Future Directions and Clinical Translation

mRNA vaccines have significant potential to reform dental implantology by enhancing osteointegration and tissue regeneration [79]. However, interpreting findings from orthopedic applications to dental practice requires addressing the unique challenges of the oral environment, such as microbial interactions and compatibility with dental materials [80]. Continued research is necessary to refine delivery systems, optimize vaccine formulations, and assess the long-term safety and efficacy of these treatments [81]. The cost-effectiveness of mRNA vaccines also needs to be carefully evaluated, as localized treatments for dental implants could pose financial barriers.

## 6. Dental Caries

**Figure 4:**
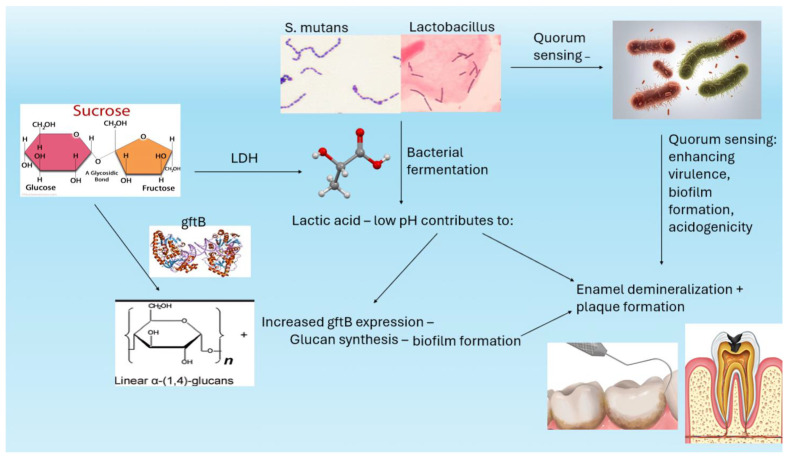
Pathogenesis of dental caries and a place for mRNA vaccines against *S. mutans* offer a promising prophylactic option and significant long-term savings. *S. mutans*, the main pathogen causing oral dysbiosis, utilizes polysaccharides such as glucans to produce lactic and other organic acids that favor tooth demineralization and plaque formation. Other bacteria such as Lactobacillus contribute to biofilm formation and communicate through “quorum sensing” mechanisms.

1. Pathogenesis overview: Dental caries is a multifactorial, biofilm-mediated, chronic oral disease where *Streptococcus mutans* (*S. mutans*) is considered the most significant etiological pathogen due to its exceptional ability to form biofilms [82]. Within the plaque biofilm, *S. mutans* utilizes polysaccharides and produces lactic acid, forming 70% of the organic acids in the biofilm [83]. These acids lead to an imbalance in remineralization and demineralization, favoring tooth demineralization, and, ultimately, dental caries [84] (Figure 4). Thereby, inhibiting biofilm formation presents a promising strategy in dental caries prophylaxis [85].
2. The Potential of RNA-Based Therapies in Caries Prevention

Recent techniques in the field of dentistry have increasingly prioritized disease prevention and the conservation of tooth structure rather than surgical treatment [86]. Early efforts, such as the 2006 development of oligodeoxyribonucleotides targeting gtfB mRNA, demonstrated the ability to reduce glucan production and biofilm formation [87]. However, limited progress in RNA-based approaches was made until recently. The 2024 study by Shung Yu introduces a groundbreaking anti-caries strategy combining *S. mutans* antisense vicK RNA (ASvicK) with Dimethylaminohexadecyl methacrylate (DMAHDM), providing a synergistic effect in biofilm reduction. ASvicK RNA specifically targets and inhibits the expression of vicK, a key gene involved in glucan synthesis, impairing the biofilm’s structural integrity and virulence [88]. Concurrently, DMAHDM disrupts the bacterial cell membrane by integrating into the lipid bilayer, increasing membrane permeability and leading to bacterial cell death. This dual-target approach significantly enhances biofilm control and cariogenicity reduction compared to single-target strategies. Additionally, this combination minimizes enamel demineralization, as evidenced by in vitro studies, demonstrating its potential to prevent caries at an early stage. This innovative approach addresses key steps in the pathogenesis of dental caries and offers a promising, targeted method for disease prevention, paving the way for RNA-based dental therapeutics.

3. The Impact and Future of mRNA Anti-Caries Vaccines

Contrary to previous beliefs of it being only a pediatric issue, it was recognized that the disease progresses in adulthood, where its incidence varies from 26% to 85% [89]. The cost-effectiveness of anti-caries vaccines, while initially high due to development and administration costs, could lead to significant long-term savings. Traditional preventive measures, such as fluoride toothpaste and regular dental visits, require ongoing effort and maintenance [90]. An anti-caries vaccine could provide sustained immunity against *Streptococcus mutans*, significantly reducing the need for continuous treatments (fillings and root canals) and lowering the risk of complications like infective endocarditis and bone loss [91]. This shift from treatment to prevention could transform oral health care by offering a proactive, long-term solution [92].

The potential of mRNA vaccines to combat dental caries is particularly promising. Emerging therapies, such as the combination of ASvicK RNA and DMAHDM, have shown efficacy in managing caries in preclinical studies by targeting bacterial virulence and metabolism [93]. These innovations highlight the transformative potential of mRNA vaccines to reduce the global burden of caries [94].

However, their clinical readiness remains in its early stages, with challenges in research, cost, and regulatory approval yet to be overcome.

Barriers to implementation include public acceptance, logistics of vaccine administration in dental settings, and concerns about antimicrobial resistance. Addressing these issues, alongside progressing preclinical findings to clinical trials, will be critical for widespread adoption [95]. If successfully developed and approved, anti-caries vaccines could significantly enhance long-term oral health, reduce treatment costs, and improve overall quality of life.

## 7. Periodontal Disease

1. Pathogenesis of Periodontal Disease: Established Mechanisms and Emerging Insight Periodontal disease (PD) is an infection-induced chronic inflammatory disease characterized by the presence of dysbiotic plaque biofilms and the progressive destruction of the tooth-supporting tissues—periodontium. The pathogenesis of PD is not solely dependent on *P. gingivalis*. Instead, its role is amplified by interactions with other pathogens, such as *F. nucleatum*, which stabilizes biofilms and enhances the pathogenicity of *P. gingivalis* as well as *Aggregatibacter actinomycetemcomitans*, *Fusobacterium nucleatum*, or *Eikenella corrodens* [96]. The pathogens disrupt host immunity (disruption of Toll-like receptor and complement function, subversion of neutrophils and macrophages, etc.) all resulting in dysbiosis, immune dysregulation, hyper-inflammation and bone resorption by matrix metalloproteinases secretion, RANKL expression, ROS accumulation, and tissue necrosis [97]. Many attempts have been made in searching for a successful treatment, but none of them have shown optimal results. Considering that inflammatory host response plays a key role in pathogenesis, some progress was achieved with anti-TNF-a and anti-IL-1 medications [98]. Nevertheless, the initial contributor to further steps of tissue destruction and inflammation is the dysbiosis and *P. gingivalis*. Therefore, the most reasonable approach is to vaccinate before colonization and the initiation of further pathological processes in the oral cavity [99].
2. The Role of Immune Disruption

Studies reveal that strain diversity among various bacterial populations could explain differences in disease progression, as less virulent strains often coexist in asymptomatic individuals [100]. Additionally, host immune disruptions contribute significantly to disease progression, with chronic inflammation being highly driven by imbalanced T-helper cell responses (Th1 and Th17) and inadequate regulatory T-cell (Treg) activity [101]. Studies like Vaernewyck et al. (2021) also demonstrated an overactive mucosal immune response, but variability in immune outcomes due to bacterial strain differences highlights the need for targeted research to refine vaccine specificity and immune modulation [102]. These insights underline the necessity of considering both microbial interactions and immune dysregulation to fully understand PD progression.

3. Host Susceptibility and Disease Progression

Host factors such as immune system competence, genetic predispositions, and environmental exposures play a critical role in PD [103]. Dysregulated immune responses, marked by chronic inflammation and insufficient resolution, are central to tissue damage [104]. Salivary secretory IgA (SIgA) has been identified as a protective factor that promotes a symbiotic relationship between the host and microbiota [105]. However, systemic conditions like diabetes or lifestyle factors such as smoking can tip the balance, exacerbating disease severity. This highlights the need for integrated research into host–microbe interactions to develop personalized strategies for prevention and treatment.

4. mRNA Vaccines: Promising Innovations for PD Management

mRNA vaccines represent a novel approach to preventing PD by targeting key pathogens and modulating immune responses. These vaccines encode antigens that elicit protective immunity, aiming to prevent biofilm formation and mitigate inflammatory damage. Preclinical studies, particularly in rodent models, have shown that mRNA vaccines targeting *P. gingivalis* antigens can reduce alveolar bone loss and inflammation by inducing strong SIgA responses. Mucosal vaccines have demonstrated greater efficacy than systemic ones, providing robust dual immunity in the oral cavity. However, challenges remain, particularly in balancing vaccine immunogenicity and inflammation. Lipid nanoparticle (LNP) formulations have successfully enhanced antigen expression but also triggered excessive cytokine responses. Refining vaccine formulations, such as by modifying mRNA signal sequences as performed in SARS-CoV-2 vaccines, may enhance immunogenicity while mitigating inflammatory side effects [106].

5. Translational Challenges and Future Directions

Despite promising preclinical results, significant hurdles remain before mRNA vaccines for PD can achieve clinical applicability. The polymicrobial nature of PD requires multivalent vaccines to comprehensively target dysbiosis [107]. Additionally, preclinical findings in larger animal models like dogs have been inconsistent, likely due to species-specific microbiota differences and the complexity of PD pathogenesis [108]. Further research should prioritize aligning vaccine designs with human pathophysiology [109]. Strategies to optimize mucosal vaccination, which has shown stronger IgA responses, are critical. Future studies must also address questions about host susceptibility and environmental factors to ensure broader applicability and effectiveness. While the complexity of PD presents challenges, the combination of multivalent vaccine formulations and mucosal administration offers a promising path forward for prevention and management [110,111,112]. A key challenge for future RNA vaccinations in periodontitis is achieving a robust immune response without triggering the inflammatory reaction that underlies the condition’s symptoms [113]. Optimizing vaccine formulations is crucial to enhance immunogenicity while avoiding one of the primary pathogenic mechanisms—a strong inflammatory response [114].

## 8. Conclusions

In conclusion, while mRNA vaccines offer substantial promise for the future of dentistry, their clinical application remains in the early stages, requiring further investigation to address key challenges. The potential of mRNA technology in dental care, especially in areas such as dental caries, periodontal disease, and dental implantology, is well established [115] (Table 1). However, critical limitations must be addressed, including the need for more robust clinical trials, the optimization of vaccine formulations for localized applications, and the development of effective delivery systems to prevent adverse immune responses [116]. Moreover, the translation of these technologies from preclinical models to human clinical settings presents several hurdles, including regulatory approval and public acceptance.

While the potential of mRNA vaccines in other medical fields provides a solid foundation for their use in dentistry, it is essential that future research focuses specifically on dental applications to ensure that the benefits outweigh the risks [117]. Ultimately, mRNA vaccines have the capacity to reshape preventive and regenerative dental practices, particularly in enhancing immune responses, preventing infection, and promoting tissue regeneration, but a comprehensive understanding of their safety, efficacy, and long-term outcomes will be necessary for their successful integration into clinical practice [118].

**Table 1:** The table provides a detailed overview of mRNA applications in dentistry, spanning regenerative medicine, oncology, and the treatment of infectious and inflammatory diseases. Key examples include mRNA encoding BMP-2 for tissue regeneration, mRNA vaccines for HPV-related cancers, antisense oligonucleotides for bacterial virulence in dental caries, and immunomodulation for periodontal disease. It also highlights the challenges in optimizing lipid nanoparticle (LNP) systems for mRNA delivery, emphasizing the need for further advancements to fully realize the therapeutic potential of mRNA-based approaches in dental medicine.

Application Field	Study Name	Study/Authors	Study Type	Key Findings	Notes
Regenerative Applications	mRNA encoding BMP-2 enhanced osteogenesis in periodontal ligament stem cells	Liu et al. (2023)—[75]	Pre-clinical/In-vitro	mRNA encoding BMP-2 enhanced osteogenesis in periodontal ligament stem cells.	Potential application for dental regeneration.
	BMP-2 mRNA accelerated healing and implant stability in rat models of dental implant failure	Zhou et al. (2021)—[76]	Pre-clinical/In-vivo	BMP-2 mRNA accelerated healing and implant stability in rat models of dental implant failure.	Demonstrates osteoinductive potential of mRNA in vivo.
HPV realated head and neck cancers	BNT113 + pembrolizumab as first-line treatment in patients with unresectable recurrent/metastatic HNSCC: Preliminary safety data from AHEAD-MERIT	Klinghammer et al. (2022)—[53]	Clinical trial	Preliminary safety data showing promising outcomes when combining BNT113 (mRNA vaccine) and pembrolizumab for unresectable metastatic HNSCC.	BNT113 targets HPV-16 E6 and E7 antigens, which are associated with HPV-related head and neck cancers (HNSCC).
Oral Cancer (non HPV related tumors)	Personalized mRNA cancer vaccines with immune checkpoint inhibitors: A promising therapeutic approach in oral cancer patients	Gheena S, Ezhilarasan D (2023)—[16]	Prelinical	The article explores the combination of personalized mRNA cancer vaccines with immune checkpoint inhibitors for oral cancer, with preclinical findings suggesting improved tumor immunity and patient outcomes.	Focus on synergy between mRNA vaccines and immune checkpoint inhibitors for oral cancer treatment.
Dental Caries	Treatment of *Streptococcus mutans* with antisense oligodeoxyribonucleotides to gtfB mRNA inhibits GtfB expression and function	Guo, Q. Y., et al. (2006)—[87]	Preclinical	Antisense oligodeoxyribonucleotides targeting gtfB mRNA inhibited GtfB expression and function in *Streptococcus mutans*	Study focused on targeting bacterial genes to reduce virulence.
	Combination of ASvicK RNA and DMAHDM inhibited biofilm formation and reduced enamel demineralization	Shung Yu (2024)—[89]	Pre-clinical/In-vitro	Combination of ASvicK RNA and DMAHDM inhibited biofilm formation and reduced enamel demineralization.	Highlights potential of RNA-based vaccines for caries management.
Periodontal Disease	Targeting Th17 cells: a promising strategy to treat oral mucosal inflammatory diseases	Wang et al., (2023)—[100]	Pre-clinical	Targeting Th17 cells reduces inflammation in oral mucosal diseases. Inhibiting Th17 cell activity can alleviate conditions like periodontal disease by modulating the immune response.	Potential therapeutic approach for treating oral mucosal inflammation, including periodontal disease.
	Mucosal Vaccination Against Periodontal Disease: Current Status and Opportunities	Vaernewyck V (2021)—[102]	Pre-clinical	Mucosal vaccination induced strong salivary IgA responses.	Suggests efficacy of mucosal mRNA vaccines in the oral cavity.
Challenges Across Applications	Development of mRNA Lipid Nanoparticles: Targeting and Therapeutic Aspects	Liu et al. (2024)—[118]	Pre-clinical/In-vitro	Particle size, surface charge, and ionizable lipids are critical in ensuring efficient mRNA delivery and stability.	Insights into optimizing LNPs for mRNA delivery
